# Soft tissue metastases of non-small cell lung cancer

**DOI:** 10.11604/pamj.2021.39.260.30740

**Published:** 2021-08-23

**Authors:** Nabil Tiresse, Hicham Souhi

**Affiliations:** 1 Military Hospital of Instruction Mohamed V, Mohamed V University, Rabat, Morocco

**Keywords:** Metastasis, soft tissuse, lung cancer, transparietal scannoguided biopsy

## Image in medicine

A 65-year-old patient, chronic smoker, accuses a dry cough with hemoptysis since 3 months with altered general condition and weight loss. The clinical examination found a firm dorsal mass in the right axillary region and another dorsal swelling in the left subscapular, other masses are also present in the right calf, root of the right thigh. Diagnosis of soft tissue metastases (STM) of non-small lung cancer was retained by scannoguided trans parietal biopsy of the right calf. Another biopsy was necessary for immunohistochemical study in order to differentiate the histologic type of non-small cell carcinoma, but the patient died by cerebral complication of neoplasia that was already at an advanced stage at the time of diagnosis. Soft tissue metastases are perceived as a sign of advanced disease and are regarded as a grave prognostic indicator. Metastases from lung cancer are microscopically indistinguishable from metastases of other cancers. Usually, they present as fast-growing solitary or multiple nodules. Soft tissue metastases represent 1.8% of lung cancer metastasis. This metastasis is a relatively rare, but not exceptional, manifestation of lung cancer. Positron emission tomography/computed tomography (PET/CT) can detect unsuspected STM to change the staging and treatment of some patients and specifies that muscle STM were primarily found in the hip and upper limb muscle, whereas subcutaneous STM were mainly distributed in the chest, abdomen, and back.

**Figure 1:**
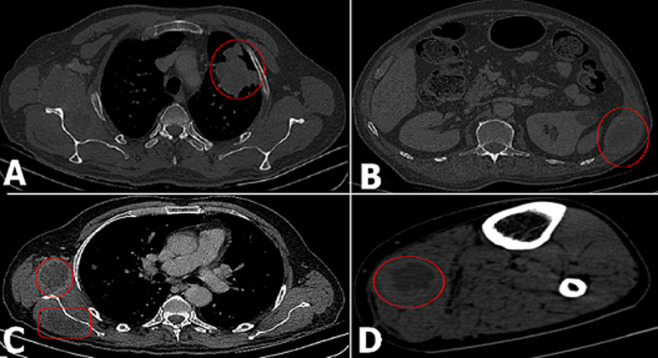
A) thoracic computed tomography (CT) showing a left upper lung mass; B) abdomino-pelvic CT showing a sympathetically maintained pain (SMP) in the left lumbar region; C) SMP shown in the right axillary region; D) CT of the right leg showing a STM in the right calf muscle

